# Some Illustrative Cases

**Published:** 1907-11-09

**Authors:** 


### Dermatology.

### SOME ILLUSTRATIVE CASES.

Among the cases presenting themselves at the skin clinic at the Prince of Wales's General Hospital, Tottenham, the following showed certain features of interest:—

### Alopecia Areata with Monilethrix.

A boy of eleven came with a condition of patchy baldness which had existed for two months, a younger brother being similarly affected. Both scalps presented numerous semi-bald areas, devoid of scaliness, but on close inspection short, broken hairs could be seen at the margins of the patches. These stumps differed from those usually met with in ringworm in that, even to the naked eye, they were thickened at their free extremities—in other words, they were club-shaped. Under the microscope their ends were seen to be frayed and brush-like, and no spores could be found. A few of the hairs were decidedly moniliform in character, having well-marked nodes and internodes. This latter condition could not have been in this case discovered without a microscopic examination, as the alternate thickenings were not sufficiently pronounced to be visible with the unaided eye. Only the elder boy appeared to be affected with monilethrix. The treatment adopted was the prescription of a stimulating paint containing one-and-a-half drachms of the oil of cinnamon in an ounce of olive oil, to be applied with a stiff brush every night, care being taken to avoid actual soreness of the skin.

### Sycosis in an Epileptic.

A man aged thirty-five suffered from "neck disease" for nine months. He had been taking for some time full doses of bromide of potassium with arsenic for epilepsy. On examination there was a collar-like band of pustular folliculitis occupying the lower part of the front of the neck only, the beard-region being free. Some of the hairs emerged from follicles that were filled with pus, and as the patient had allowed the hair to grow on the affected part only, all else being clean shaven, he presented a somewhat curious appearance. Mercurial ointments and other antiseptics had been tried without much success. A hair was taken for examination, and was found to be free from spores or mycelium; it did not come out very easily, and it was not brittle. A dilute cyllin lotion was ordered for bathing the area twice daily, and an ointment containing 25 minims of ichthyol to the ounce of dilute ammoniated mercury ointment. The condition was not considered to be due to bromides, as it was strictly localised to that part of the neck where the upper margin of the collar might press, and there were no pustular lesions elsewhere.

### Dysidrosis and Bromidrosis Pedum.

An errand-boy, aged fourteen, came with very bad sweating feet, which he had had for three weeks. They were now so sore that walking was impossible, and the odour was very offensive. The skin of the interdigital clefts and just beneath the toes was sodden, exfoliating in parts, and exuding sero-pus, secondary infection with pyogenic organisms having obviously occurred. The condition somewhat resembled scabies, but no other parts were affected, and itching had never been a marked feature. He was given a weak cyllin lotion (1 per cent.), to be put into a foot-bath of warm water, in which he was directed to soak the feet twice a day for a quarter of an hour, and after gentle drying to powder the affected parts with boracic powder containing a little iodoform (one part in twenty). When the acute condition had somewhat subsided, the cyllin lotion was changed to one of formalin (2 per cent.). The boy made a good recovery.

